# Serological responses to rotavirus NSP2 following administration of RV3-BB human neonatal rotavirus vaccine

**DOI:** 10.1080/21645515.2018.1467202

**Published:** 2018-05-31

**Authors:** Daniel Cowley, Daniel Pavlic, Nada Bogdanovic-Sakran, Karen Boniface, Carl D. Kirkwood, Julie E. Bines

**Affiliations:** a Enteric Virus Group, Murdoch Children's Research Institute, Parkville, VIC, Australia; b Rotavirus Program, Murdoch Children's Research Institute, Parkville, VIC, Australia; c Department of Paediatrics, The University of Melbourne, Parkville, VIC, Australia; d Department of Gastroenterology and Clinical Nutrition, Royal Children's Hospital, Parkville, Victoria, Australia

**Keywords:** Rotavirus, RV3-BB, vaccines, neonates, diarrhoea, serological response

## Abstract

Serum rotavirus IgA responses are an imperfect non-mechanistic correlate of protection, and the lack of an accurate serological marker is a challenge to the development of new rotavirus vaccines. Serological responses to rotavirus NSP2 occur following wild-type infection; however, it is unknown if serological responses to NSP2 occur following administration of rotavirus vaccines. The phase IIa immunogenicity trial of RV3-BB provided an opportunity to investigate the serological responses to NSP2 following vaccination. Healthy, full-term babies (n = 96) were previously recruited as part of a phase IIa safety and immunogenicity trial in Dunedin, New Zealand between January 2012 and April 2014. Participants received three doses of oral RV3-BB vaccine with the first dose given at 0–5 days after birth (neonatal schedule), or the first dose given at about 8 weeks after birth (infant schedule), or to receive placebo (placebo schedule). Serum IgA and IgG antibody responses to total RV3-BB and NSP2 protein (RV3-BB) were assessed using ELISA. Despite significant serum IgA response against total RV3-BB, we were unable to demonstrate a significant serological response to NSP2 in participants receiving RV3-BB when compared to placebo. Heterotypic antibodies against multiple NSP2 genotypes were detected following RV3-BB vaccination. Our data demonstrates that while serological responses to NSP2 were detectable in a subset of participants, it is a less useful marker when compared to total rotavirus serum IgA response.

## Introduction

Rotavirus is the most common cause of severe diarrhoea in children under 5 years of age. Three vaccines, Rotarix® (GSK, Rixensart, Belgium), RotaTeq® (Merck, Whitehouse Station, USA) and ROTAVAC (Bharat Biotech, Hyderabad, India) are WHO prequalified and available in more than 100 countries. New rotavirus vaccines are in development to address concerns regarding reduced efficacy in low-income countries^1^^,^^2^ and supply.^3^ One candidate is the human neonatal vaccine RV3-BB, based on a naturally attenuated neonatal G3P[6] rotavirus strain. The intrinsic characteristics of the RV3-BB vaccine makes it ideal for a birth dose administration schedule which may have benefits to provide early protection in low-income settings.^4^^,^^5^

Following primary infection, IgM, IgG and IgA antibodies against rotavirus appear in serum and mucosal secretions.^6^ Several rotavirus structural proteins are immunogenic in humans, including the viral proteins (VP) VP6, VP2, VP7 and VP4.^7-10^ The non-structural proteins (NSP) NSP2 and NSP4 have also been shown to be immunogenic.^7^^,^^9^^,^^11^^,^^12^ A significant correlation between total rotavirus serum IgA, using the lysate of virus-infected cells as the capture antigen, and protection following natural infection and vaccination has been identified.^13^^,^^14^ However, total rotavirus serum IgA titres are an imperfect correlate of protection and may have limited utility in low income settings.^15^ The clinical development of new vaccine candidates poses a challenge as placebo-controlled studies may be unethical due to the availability of effective vaccines. Non-inferiority or superiority studies for new vaccines with clinical endpoints require large sample sizes and are expensive to conduct.^15^ Therefore, there is a need to identify new immunological markers which can predict protection after rotavirus vaccination.

The non-structural protein NSP2 is a component of rotavirus replication intermediates and accumulates in cytoplasmic inclusions (viroplasms), sites of genome RNA replication and the assembly of sub-viral particles.^16^^,^^17^ Antibodies to NSP2 rise to high titre after wild-type rotavirus infection and are boosted after re-infection.^11^ NSP2 specific antisera can inhibit rotavirus replication *in vitro* leading to limited virus replication.^18^ This suggests immune responses to NSP2 contribute to protection against disease by blocking the functions of NSP2 in viral replication and might be a marker of vaccine protection. However, it is unknown if serological responses to NSP2 occur following administration of rotavirus vaccines. The phase IIa immunogenicity trial of RV3-BB in New Zealand^5^ provided an opportunity to investigate the serological responses to NSP2 following vaccination. The aim of this current study was to determine if serum antibody responses to the NSP2 protein occur following RV3-BB vaccination. We also sought to determine if antibody responses to the NSP2 protein are a useful marker to understand serological responses following vaccination.

## Results

### The proportion of participants with seroconversion to total RV3-BB are higher when compared to RV3-BB NSP2 protein

The cumulative serological responses (>three fold rise above baseline) to total RV3-BB have previously been reported^5^ and are included here to allow comparison with the anti-NSP2 responses. In the neonatal vaccine schedule, serum IgA responses to total RV3-BB were detected in 19/30 (63.3%) participants, compared with 3/32 (9.3%) in placebo (difference in proportions 0.54, 95% CI 0.28-0.71; *p* < 0.0001). In the infant vaccine schedule, serum IgA responses were detected in 20/27 (74.1%) participants compared with 8/32 (25%) in the placebo (difference in proportions 0.49, 95% CI 0.24-0.66; p = 0.0002). In contrast, the proportion of participants with serum IgA responses to RV3-BB NSP2 protein were not significantly different between the vaccine or placebo groups. In the neonatal schedule, serum IgA responses to NSP2 were detected in 11/30 (36.7%) participants compared with 9/32 (28.1%) in placebo (difference in proportions 0.08, 95% CI -0.16 to 0.32, p = 0.589). In the infant vaccine schedule, serum IgA responses to NSP2 were detected in 9/27 (33.3%) participants compared to 7/32 (21.9%) in the placebo (difference in proportions 0.11, 95% CI -0.13 to 0.35; p = 0.386). A subset of participants who responded the RV3-BB NSP2 protein also demonstrated IgA serological responses to against other NSP2 proteins including the RV5 (N2), SA11 (N5) and RV4 (N1), demonstrating the responses were heterotypic (Table 1).

**Table 1.** Serological responses to NSP2 proteins of different rotavirus strains following administration of RV3-BB or placebo.

		NSP2 protein (genogroup)			
Participant #	Schedule	RV3-BB (N1)	RV4 (N1)	RV5 (N2)	SA-11 (N5)
008	Neonatal	**+**	**+**	**+**	**+**
071	Neonatal	**+**	**+**	−	−
046	Neonatal	−	−	−	−
043	Neonatal	−	−	−	−
045	Infant	**+**	−	**+**	**+**
086	Infant	**+**	−	−	**+**
050	Infant	−	−	−	−
075	Infant	−	−	−	−
056	Placebo	**+**	−	−	**+**
038	Placebo	**+**	**+**	**+**	**+**
014	Placebo	−	−	−	−
011	Placebo	−	−	−	−

Neonatal and Infant schedule participants received RV3-BB vaccine according to the dosing schedule described in Material and methods. (+) > 3x rise in antibody titre; (−) no serological response.

Serum IgG responses were limited against total RV3-BB and NSP2 protein irrespective of the vaccination schedule. In the neonatal vaccine schedule, serum IgG response to total RV3-BB and NSP2 were detected in 3/30 (10%) and 1/30 (3.3%) and participants respectively. No serum IgG responses to total RV3-BB or NSP2 protein were detected in the placebo group. In the infant vaccine schedule, serum IgG responses to total RV3-BB were detected in 4/27 (14.8%) participants and none in placebo group. Serum IgG responses to NSP2 were detected in 2/27 (7.4%) participants in the infant vaccine schedule and 1/32 (3.1%) in the placebo group.

### Limited utility of serum IgA or IgG antibodies against RV3-BB NSP2 protein as marker of serological response

**Figure 1.**
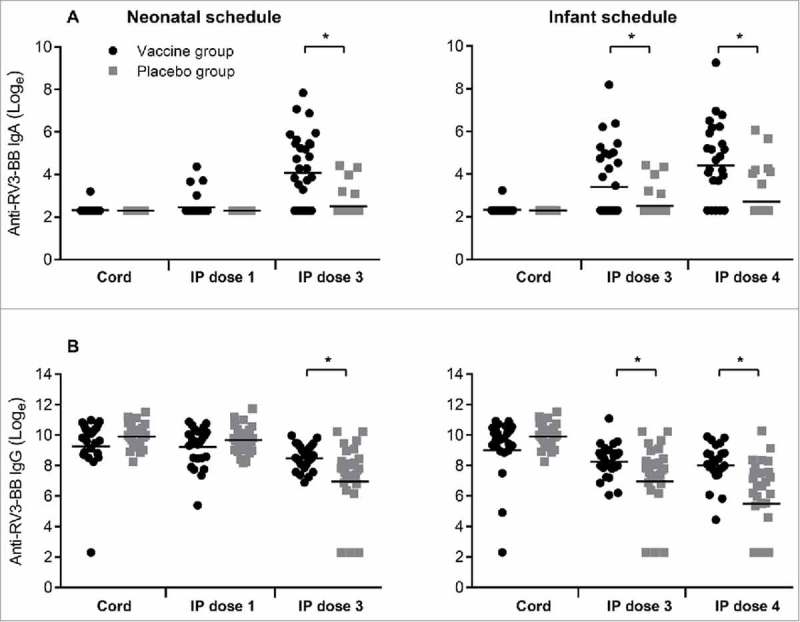
Titres of Anti-RV3-BB antibodies in cord blood collected at birth and in serum following dosing with RV3-BB or Placebo according to the schedule described in the Materials and methods. Serum IgA (A) and serum IgG (B) in Neonatal and Infant schedules by dose and compared to Placebo. Data points represent individual serum samples, horizontal lines represent the geometric mean titre (GMT). Data is transformed onto a natural log scale. *Significant difference between geometric mean.

Following three doses of vaccine, serum IgA titres to total RV3-BB were significantly higher when compared to the placebo group, in both the neonatal (GMT 80.6 vs 12.9, geometric ratio 6.22, 95% CI 3.19 to 12.13) and infant schedules (GMT 113.2 vs 17.5, geometric ratio 6.46, 95% CI 2.91 to 14.3) (Fig. 1A). Similarly, serum IgG titres were significantly higher when compared to the placebo group following three doses of RV3-BB in both the neonatal (GMT 5037.5 vs 1612.2, geometric ratio 3.12, 95% CI 1.69 to 5.74) and infant schedules (GMT 3326.7 vs 435.6, geometric ratio 7.63 95% CI 3.40 to 17.14) (Fig. 1B).

**Figure 2.**
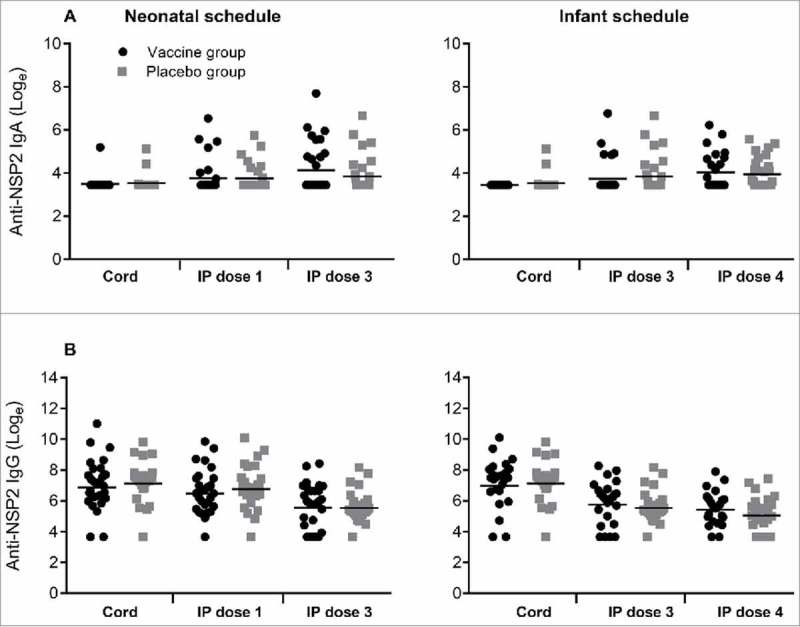
Titres of Anti-NSP2 antibodies in cord blood collected at birth and in serum following dosing with RV3-BB or Placebo according to the schedule described in the Materials and methods. Serum IgA (A) and serum IgG (B) in Neonatal and Infant schedules by dose and compared to Placebo. Data points represent individual serum samples, horizontal lines represent the geometric mean titre (GMT). Data is transformed onto a natural log scale. *Significant difference between geometric mean.

In contrast, antibody titres to RV3-BB NSP2 protein were not significantly different in the vaccine or placebo groups irrespective of dose or schedule (Fig. 2A). Following three doses of vaccine the IgA titre to NSP2 was similar when compared to the placebo group in both the neonatal (GMT 71.1 vs 50.4, geometric ratio 1.41, 95% CI 0.81 to 2.45) and infant schedules (GMT 61.3 vs 54.8, geometric ratio 1.11, 95% CI 0.71 to 1. 75). The IgG titres to NSP2 were high in all cord blood samples, GMT range 1186 -1416 (Fig. 2B). In both neonatal and infant vaccine schedules there was a decrease in IgG titre following three doses of RV3-BB, with no evidence of boosting. There were no significant differences in IgG titres between vaccine or placebo groups in any schedule or dose.

## Discussion

Following wild-type infection antibodies to NSP2 rise, are boosted after re-infection and detectable in convalescent serum.^7^^,^^9^^,^^11^ Antibodies to NSP2 inhibit rotavirus replication *in vitro* by binding to a surface exposed epitope which is conserved across different NSP2 genotypes.^18^ These reports suggest that immune responses NSP2 may be a useful mechanistic correlate of protection following vaccination. We were unable to demonstrate a significant serological response to NSP2 in participants receiving RV3-BB vaccine when compared to placebo. However, in a subset of participants we demonstrated serological responses to multiple NSP2 genotypes following RV3-BB vaccination, similar to previously described following wild-type infections.^11^ It is unclear if this lack of a significant NSP2 serological response following vaccination is unique to the RV3-BB vaccine or is similar with other rotavirus vaccines, as we are unaware of other reports describing anti-NSP2 serological responses post vaccination.

Rotavirus-specific IgG antibodies in the cord blood are derived from transplacental transfer from maternal circulation and may have an inhibitory effect on the immunogenicity of rotavirus vaccines.^19^^,^^20^ As previously reported,^21^ the high titre of maternal derived rotavirus-specific IgG antibodies made it difficult to detect IgG serological responses following vaccination in neonates. However, we demonstrated high titres of anti-NSP2 IgG antibodies in the cord blood samples from most participants. Anti-NSP2 IgG and IgA responses occur in a high proportion of children following symptomatic primary infection,^7^^,^^9^^,^^11^ suggesting that anti-NSP2 serum responses are important for protection from disease.^11^ We identified no difference in anti-NSP2 IgG titre between vaccine and placebo groups, with the titre declining at similar rates in each group. This suggests the anti-NSP2 IgG antibodies detected in serum samples post vaccination were maternally derived and not generated in response to RV3-BB. We have previously demonstrated that cord blood anti-rotavirus IgG did not impact the serum IgA response or stool excretion after 3 doses of RV3-BB or after one dose.^22^ This suggests limited impact of the anti-NSP2 IgG antibodies on the RV3-BB vaccine immune responses. However, the role of these maternally derived anti-NPS2 IgG antibodies in the immunity to wild-type rotavirus infections in the newborn remains to be elucidated.

### Study limitations

Using total RV3-BB as the capture antigen in ELISA, the proportion of participants with IgA serum conversion and IgA and IgG GMTs were significantly higher in both the neonatal and infant schedules when compared to placebo. The majority of antibodies present in serum are directed against the capsid proteins VP6, VP2, VP7 and VP4.^7^^,^^8^^,^^10^ It is likely that multiple antigens increase sensitivity for detecting serological responses when compared to a single antigen such as NSP2. The lack of significant serological response to NSP2 may also suggest that this protein is less immunogenic when compared to other RV3-BB antigens.

NSP2 is not packaged in virions and is only expressed following viral entry and replication.^23^ Therefore, to be immunogenic following vaccination, there must be replication *in vivo*. We have previously demonstrated replication of RV3-BB following vaccination and shedding in >70% of participants, similar to other rotavirus vaccines.^24^^,^^25^ However, since rotavirus vaccines such as RV3-BB are attenuated, stool viral loads and duration of shedding are lower when compared to wild-type infections.^25^^,^^26^ Limited *in vivo* replication may explain the lower than expected serological responses to NSP2 following RV3-BB vaccination when compared to previous studies examining serum from children hospitalized with primary rotavirus infection.^7^^,^^11^ Similarly, it is possible NSP2 serological responses detected in the vaccine and placebo groups were due to undetected wild-type infections and not the RV3-BB vaccine.

### Conclusions

Our findings show that there are limited serological responses to NSP2 protein following vaccination with RV3-BB. The antibody responses to the NSP2 protein are less useful marker of serological responses following vaccination when compared with total rotavirus serum IgA responses.

## Materials and methods

### Study design and serum collection

The design, recruitment and demographic characteristics of the Phase IIa safety and immunogenicity clinical trial of RV3-BB vaccine has been previously described.^5^^,^^25^ Briefly, a randomised, double-blind, 3-arm, placebo-controlled trial in 96 participants was conducted at Dunedin Hospital, New Zealand between January 2012 and April 2014. Eligible participants were randomly assigned into one of three treatment groups; neonatal vaccine schedule group, infant vaccine schedule group, or placebo. The final analysis included 30 participants in the neonatal schedule, 27 in the infant schedule and 32 in the placebo schedule. The investigational product (IP) consisted of RV3-BB vaccine (∼8.3 × 10^6^ FCFU/ml) or Placebo (cell culture medium, DMEM), prepared by Meridian Life Sciences (Memphis, TN). Participants received four oral doses of IP (either RV3-BB vaccine or placebo) according to the treatment group randomisation schedule, with doses administered at 0–5 days (dose 1), 8 weeks (dose 2), 14 weeks (dose 3) and 22 weeks of age (dose 4). In the neonatal arm, dose 1, 2 and 3 consisted of RV3-BB vaccine whilst dose 4 was placebo. In the infant arm, dose 1 was placebo whilst dose 2, 3 and 4 consisted of RV3-BB vaccine. In the placebo arm all four doses given were placebo. Cord blood was collected from the umbilical cord and subsequent serum samples were collected from all participants 28 days after IP dose 1, 28 days after IP dose 3, and 28 days after IP dose 4. Serum samples were stored at −70°C until analysed.

### Expression and purification of recombinant NSP2 protein

Recombinant NSP2 protein was generated from RV3-BB, RV4 (G1P[8]) and RV5 (G2P[4]). Viral RNA was extracted using the QIAamp® Viral RNA Mini Kit (QIAGEN, Cat# 52906). Rotavirus gene segment 8, was amplified using high fidelity RT-PCR (TaKaRa Cat#R022A). The primer sequences used for RV3-BB were for 5′-CCGAAACCATGGCTGAGCTAG-3′ and rev 5′CGGAGATCTAACTCCAACATGTGAGAC; RV4 for 5′-CCGAAACCATGGCTGAGCTAG-3′ and rev 5′ CGGAGATCTAATTCCAACGTGCGAAAC-3′; RV5 for 5′-CCGAAACCATGGCTGAGCTAG-3′ and rev 5′-CGGAGATCTAATTCCTACTTGAGAAAC-3′. PCR products were cloned into pQE60 as previously described.^17^ Recombinant NSP2 protein was expressed in Escherichia coli M15[pREP4] cells and purified using nickel-nitrile acetate (Ni-NTA) agarose columns according to manufacturer's instructions (QIAGEN Cat# 30210) and previously described.^17^ The NSP2 proteins were evaluated by electrophoresis on 10% sodium dodecyl sulfate–polyacrylamide gels (SDS–PAGE) according to Laemmli [1970] and Coomassie blue R-250 staining and were purified to near homogeneity when compared to mock *E. coli* M15[pREP4] extracts. The identity of expressed proteins were confirmed by western blot using anti-SA11 rabbit polyclonal serum. M15 cells containing pQE60-SA11 expression vector were a gift from John Patton, SA11 NSP2 protein was expressed and purified as described above.

### Enzyme-linked immunosorbent assay of NSP2 specific IgA and IgG antibodies

The serological assays to assess serum immune responses against NSP2 protein were modified from a method previously described.^11^ Microtiter plates (Nunc Maxisorp, Cat# 44-2404-21) were coated with 100 µl/well (4 µg/ml) of NSP2 protein in 0.06 M carbonate-bicarbonate buffer (pH 9.6), and incubated at 4°C for 6 h. Plates were washed and blocked with 0.5% (w/v) Casein-PBST overnight at 4°C. Serum samples at a single dilution (1:20 for IgA and 1:100 for IgG) were added and incubated at 37°C for 1.5 h. The bound antibodies were bound with biotinylated anti-human IgA or IgG (Jackson ImmunoResearch Laboratories Cat #309-065-011 and 309-065-008) (diluted 1:8,000 for IgA and 1:32,000 for IgG) incubated at 37°C for 1.5 h. The antigen–antibody complexes were detected with streptavidin-horseradish peroxidase (diluted 1:200,000) (Invitrogen, Cat# SNN1004). The colour reaction was developed using 3,3,5,5-tetramethylbenzidine (TMB) at 0.1 mg/ml diluted in 0.1 M Na-acetate buffer with 0.036% (v/v) H_2_O_2_. The reaction was stopped with 2 M H_2_SO_4_. The optical density was measured at 450 nm using an ELISA plate reader (Titertek Multiskan, Flow). The dilutions of antibody and conjugates were made in PBS-Tween containing 0.5% casein. Streptavidin-horseradish peroxidase was diluted in PBS-Tween only. All wash steps were performed four times using an automatic 96 well plate washer (Thermofisher) using PBS-Tween 20.

All samples were assayed in duplicate against wells coated with NSP2 antigen or carbonate buffer alone. The mean corrected absorbance of NSP2 well was calculated by subtraction of the carbonate buffer alone wells. Concentrations of NSP2 specific IgA and IgG were measured with a standard curve generated from known positive serum samples arbitrarily assigned a titre of 20,000 units (U/mL). The lower detection limit of the IgA assay was 62.5 U/mL and the lower detection limit of the IgG assay was 78 U/mL. If IgA or IgG antibodies against NSP2 were not detected in a sample, the concentration assigned corresponded to 50% of the lower limit (i.e., 31.25 U/mL for IgA and 39 U/ml for IgG).

### Statistical methods

Serological responses were defined as >three fold rise above baseline, defined as cord blood for the neonatal schedule comparison and serum collected after IP dose 1 for the infant schedule comparison. The proportion of participants with a serological response following each dose of IP were presented cumulatively. Differences in the proportion of participants with a serological response by dose and vaccine schedule were assessed using Fisher's exact test, with the 95% confidence intervals estimated by the Newcombe/Wilson method. Serum IgA and IgG antibody concentrations were log transformed prior to analysis. Differences antibody titre were presented as geometric mean ratio and the 95% confidence intervals for the geometric mean ratio.
